# Research progress and clinical translation prospects of multiple urinary biomarkers in non-invasive diagnosis of urothelial carcinoma

**DOI:** 10.3389/fonc.2026.1984066

**Published:** 2026-10-08

**Authors:** Shuxin Li, Lei Wang, Baoshan Gao

**Affiliations:** Department of Urology, The First Hospital of Jilin University, Changchun, China

**Keywords:** cell-free DNA, DNA methylation, exosomes, liquid biopsy, microRNA, multi-omics, urinary biomarkers, urothelial carcinoma

## Abstract

Urothelial carcinoma is a common urinary malignancy with high incidence, recurrence, and mortality. Early diagnosis is pivotal for improving patient outcomes. Current standards have important limitations: cystoscopy, although the cornerstone of diagnosis and surveillance, is invasive and costly, while cytology has limited sensitivity for low-grade or early-stage lesions. Urine, as a liquid biopsy, offers a non-invasive and repeatable approach for sampling urogenital pathology, with the potential for longitudinal monitoring through serial collection. The review covers urinary multi-omics biomarkers, including cfDNA/methylation, miRNAs, extracellular vesicles (EVs), and proteomic/metabolomic signatures. We summarize their performance in early detection, staging, and postoperative monitoring. Integrated multi-analyte models, including true multi-omics assays and multiplex single-omics panels, generally outperform single biomarkers, with some panels achieving >90% sensitivity and >95% specificity in prospective cohorts. Despite progress, several challenges remain: standardization, large-scale prospective validation, and regulatory/reimbursement barriers. We conclude that urinary multi-omics diagnostics are promising complementary tools that may optimize surveillance and risk stratification. Still, limited prospective validation, lack of standardization, and the persistent risk of false-negative results constrain adoption. On current evidence, they are not positioned to replace cystoscopy, which remains the cornerstone of diagnosis and surveillance, particularly in non-muscle-invasive bladder cancer (NMIBC). However, their most realistic and immediate clinical role is as a triage tool to optimize the selective use of cystoscopy—particularly for risk-stratifying patients with hematuria, adjudicating equivocal cytology, and potentially reducing the frequency of invasive surveillance in low-risk NMIBC patients.

## 1 Introduction

Urothelial carcinoma is one of the most common malignant tumors of the urinary system. Its clinical management has long faced severe challenges because of its high incidence, recurrence rates, and mortality; early diagnosis is undoubtedly an important factor in improving patient prognosis (1, 2). However, the current standardized diagnostic pathway, which relies on cystoscopy and urinary cytology, has significant limitations in practice: although transurethral cystoscopy is considered the “gold standard,” it is invasive, often causes pain and discomfort, and carries a risk of infection. Furthermore, it is costly and operator-dependent, limiting its suitability for large-scale population screening and motivating the development of non-invasive tools that may reduce the frequency of invasive surveillance in selected low-risk patients (1, 2). However, cystoscopy remains a cornerstone of surveillance for non-muscle-invasive bladder cancer. Although urinary cytology has high specificity, its sensitivity to low-grade and early-stage lesions is insufficient, resulting in persistently high rates of missed diagnoses (1, 2). These inherent shortcomings urgently call for a new, non-invasive, reproducible, and highly sensitive diagnostic strategy to reshape the paradigm of early detection and comprehensive management of urothelial carcinoma.

Against this backdrop, urine—an ideal sample for “liquid biopsy”—offers unique biological advantages and clinical suitability. Urine contains metabolites, proteins, shed cells, and free nucleic acids, and may provide serial information reflecting the local pathological state of the urinary system (3). Collection is completely non-invasive and simple; patients can collect repeated samples on their own, greatly improving compliance with long-term monitoring (3–5). In recent years, by detecting circulating tumor cells, cell-free DNA/RNA, extracellular vesicles, and various protein metabolites in urine, researchers have effectively mapped the molecular profile of urothelial carcinoma (6). Notably, the sensitivity of urinary cell-free tumor DNA in detecting cancer-related mutations and epigenetic alterations is now comparable to blood-based cell-free DNA liquid biopsies, strongly validating this sample type for detecting urological tumors (5). Because it is non-invasive, repeatable, and suitable for longitudinal sampling, urine testing applies not only to urological tumors but also shows strong promise for auxiliary diagnosis and recurrence prediction in various solid tumors (4, 7).

The key driver for translating this potential into clinical application will be generating robust prospective evidence and health-economic analyses, enabled by advances in multi-omics technologies. Integrated analysis of multidimensional information—including genomic, epigenomic, transcriptomic, proteomic, and metabolomic data—can significantly improve the sensitivity and specificity of early cancer detection and provide a more comprehensive assessment of tumor molecular heterogeneity and its changes over time (3, 8, 9). Urine is a pooled sample containing material from multiple cell populations and tissues. Therefore, urinary analyses reflect bulk molecular signals rather than directly resolving the spatial distribution of intratumoral molecular features. Compared with single biomarkers or single-omics strategies, integrating multimodal data from multiple sources of circulating analytes provides complementary, more robust biological signals. It demonstrates superior efficacy in early tumor screening and longitudinal monitoring (10, 11). Currently, research on urinary multi-omics biomarkers has expanded to include multiple levels, including DNA mutations, methylation modifications, non-coding RNA, and protein and metabolic characteristics, making it an emerging frontier in non-invasive diagnostics. In the future, combining high-throughput multi-omics data with advanced analytical algorithms is expected to drive urine testing technology toward timely, standardized, and intelligent applications. This will ultimately facilitate the effective translation of scientific discoveries into routine clinical workflows, thereby playing an increasingly important role in precision medicine and personalized, comprehensive management of urothelial carcinoma.

However, despite the wealth of encouraging research data, a critical gap persists between the reported diagnostic performance of urinary multi-omics biomarkers and their actual adoption in routine clinical practice. This review is therefore organized around three questions: (1) How well do individual urinary biomarker classes perform in early detection, and which technological platforms enable their clinical application? (2) What is the added value of integrating multiple omics layers over single-analyte approaches, particularly in staging and postoperative monitoring? (3) Why do major clinical guidelines remain conservative despite promising research evidence, and what barriers must be addressed to translate these technologies into clinical practice?

### 1.1 Literature search strategy and review methodology

Given the broad, multi-disciplinary scope of this field and the marked heterogeneity in study designs, omics platforms, detection methods, and outcome measures across published reports, a quantitative meta-analysis was not feasible. Instead, we adopted a narrative review approach to synthesize the evidence, with the explicit aim of critically examining translational gaps and outlining a roadmap for clinical integration.

To ensure a comprehensive and reproducible capture of relevant literature, we performed a structured search in three major biomedical databases—PubMed/MEDLINE, Web of Science, and Embase—from inception to September 15, 2026. We executed the final search on September 15, 2026, and last updated it on that date during revision. The search strategy combined controlled vocabulary (MeSH terms) and free-text keywords, including: “urinary bladder neoplasms”/”urothelial carcinoma,” “urine”/”urinary,” “liquid biopsy,” “cell-free DNA”/”cfDNA,” “DNA methylation,” “microRNA”/”miRNA,” “exosomes”/”extracellular vesicles,” “proteomics,” “metabolomics,” and “multi-omics.” The core search framework was: (urinary bladder neoplasms OR urothelial carcinoma) AND (urine OR urinary) AND (biomarker OR liquid biopsy OR cfDNA OR methylation OR miRNA OR exosome OR proteomics OR metabolomics), with database-specific syntax adaptations.

Two researchers independently screened titles and abstracts, followed by full-text reviews. Because this review addresses not only clinical performance but also technical platforms, guideline recommendations, and translational barriers, we included multiple categories of evidence: (1) original clinical studies involving patients with urothelial carcinoma and urine as the sample type, evaluating diagnostic, screening, or postoperative monitoring performance of urinary biomarker assays (single- or multi-marker, single- or multi-omics); (2) clinical practice guidelines and guideline-related documents from major urological societies (EAU, AUA, NCCN); (3) methodological and technical studies on urinary biomarker enrichment, detection, and analytical platforms; and (4) systematic reviews, meta-analysis, and narrative reviews used for context, comparison, or identification of evidence gaps. We excluded conference abstracts, case reports, editorials, and non-peer-reviewed literature. We did not use non-clinical studies as primary evidence for clinical performance, but included them when they addressed technical, methodological, or translational aspects. For clinical performance estimates, priority was given to original clinical studies, particularly prospective and multicenter studies. We manually screened the reference lists of included articles to identify important studies missed by the electronic search.

Because studies varied substantially in omics platforms, detection methods, patient populations, and outcome measures, we used narrative synthesis rather than a quantitative meta-analysis. We categorized and summarized findings by biomarker molecular type and by clinical application. This approach allowed us to present the current state of evidence, critically appraise study limitations, compare guideline recommendations, and identify key translational barriers—a perspective that a quantitative synthesis alone would not capture.

### 1.2 What this review adds to the existing literature

In this review, “multi-omics” refers to the integration of two or more distinct molecular omics layers (e.g., genomic mutation and epigenomic methylation, or proteomic and metabolomic data). “Multi-marker” or “multiplex” refers to the simultaneous assessment of multiple markers within a single omics layer. “Multi-analyte” or “composite” is used as an umbrella term for any combination of multiple biomarkers, regardless of omics layer. “Multimodal” refers to integration across different data types, such as imaging, pathology, omics, and clinical variables. We use these distinctions consistently throughout the review.

Previous reviews have provided valuable syntheses of urinary biomarkers in urothelial carcinoma, often focusing on individual biomarker classes such as cfDNA, miRNAs, exosomes, or cataloging diagnostic performance. This review is not intended to duplicate those efforts. Instead, it emphasizes three clinically oriented questions: how individual urinary biomarker classes perform in early detection; whether multi-analyte or multi-omics integration adds value in staging and postoperative monitoring; and why guideline adoption remains limited despite promising research findings. Within this framework, we aim to contribute by comparing the positions of the EAU, AUA, and NCCN guidelines side by side, examining pre-analytical and analytical standardization as translational barriers rather than merely technical details, and distinguishing analytical validity, clinical validity, and clinical utility, with particular attention to the latter, which remains the least developed part of the evidence base. A recurring theme is that the choice between exosomal and non-exosomal analytes reflects a trade-off between biological specificity and operational simplicity (**Figure 1**). We present these points as our emphasis and interpretation, rather than as claims that previous reviews have overlooked them.

**Figure 1:**
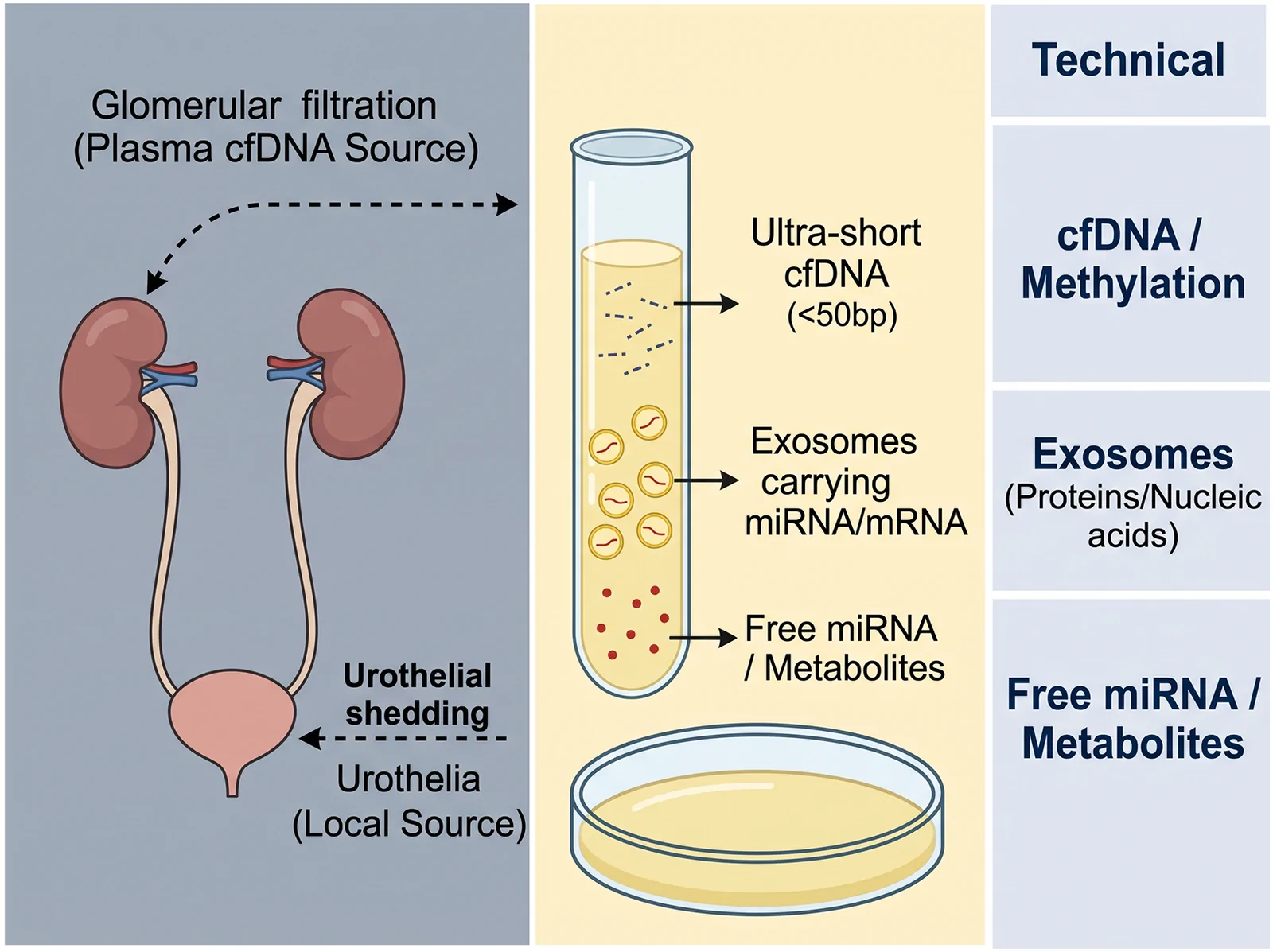
Overview of urinary multi-omics biomarker sources and classification. This schematic illustrates the major sources of urinary biomarkers and their corresponding molecular classes. The left panel depicts the anatomical origins of urinary biomarkers, including glomerular filtration of plasma-derived cell-free DNA (cfDNA) from the kidneys and direct shedding from the urothelial lining of the bladder and upper urinary tract. The middle panel shows a urine sample containing diverse analytes: ultrashort cfDNA fragments (<50 bp, depicted as dashed lines) and extracellular vesicles (30–150 nm vesicles carrying miRNAs, mRNAs, and proteins). The term “exosome” is reserved for the endosomal-origin EV subtype; endosomal origin was not demonstrated in this schematic. The right panel classifies these analytes into three major categories: cfDNA/methylation markers, exosomal cargoes (nucleic acids and proteins), and free miRNAs/metabolites. This framework highlights the molecular heterogeneity of urine as a liquid biopsy substrate and provides a structural roadmap for technical and clinical discussions in subsequent sections. cfDNA, cell-free DNA; miRNAs, microRNAs; mRNAs, messenger RNAs.

## 2 Molecular types, enrichment and identification, and detection technology systems for urinary multi-omics biomarkers

As an ideal medium for liquid biopsy, urine contains biological information characterized by high molecular heterogeneity and omics complementarity. In recent years, improved detection sensitivity and innovations in separation technologies have shaped a multi-omics technical framework centered on urinary cell-free DNA (cfDNA), microRNAs (miRNAs), exosomes and their contents, and proteins and metabolites. Rather than exhaustively cataloging all technical variants, this section focuses on the key technological platforms that have reached sufficient maturity for clinical evaluation, with particular emphasis on pre-analytical and analytical bottlenecks that directly impact translational feasibility—including detection sensitivity for ultra-short cfDNA fragments, standardization gaps in exosome isolation, and the lack of reference materials for cross-platform comparability.

This section focuses on the analytical platforms that enable urinary multi-omics analysis, rather than on clinical performance in a specific tumor type. Several platforms were initially developed or validated in other genitourinary or non-genitourinary malignancies, including prostate cancer, renal cell carcinoma, kidney allograft injury, and non-small-cell lung cancer. We cite such studies only as technical proof-of-concept or methodological examples to illustrate analytical feasibility; we do not use them to estimate diagnostic sensitivity, specificity, or clinical utility in urothelial carcinoma. Where available, we prioritize studies performed in urine from patients with urothelial carcinoma or bladder cancer. Evidence derived from other tumor types is explicitly identified as such throughout this section. **Figure 2** summarizes which platforms have been directly evaluated in urothelial carcinoma versus those supported only by cross-tumor technical evidence, and Section 3 discusses this.

**Figure 2:**
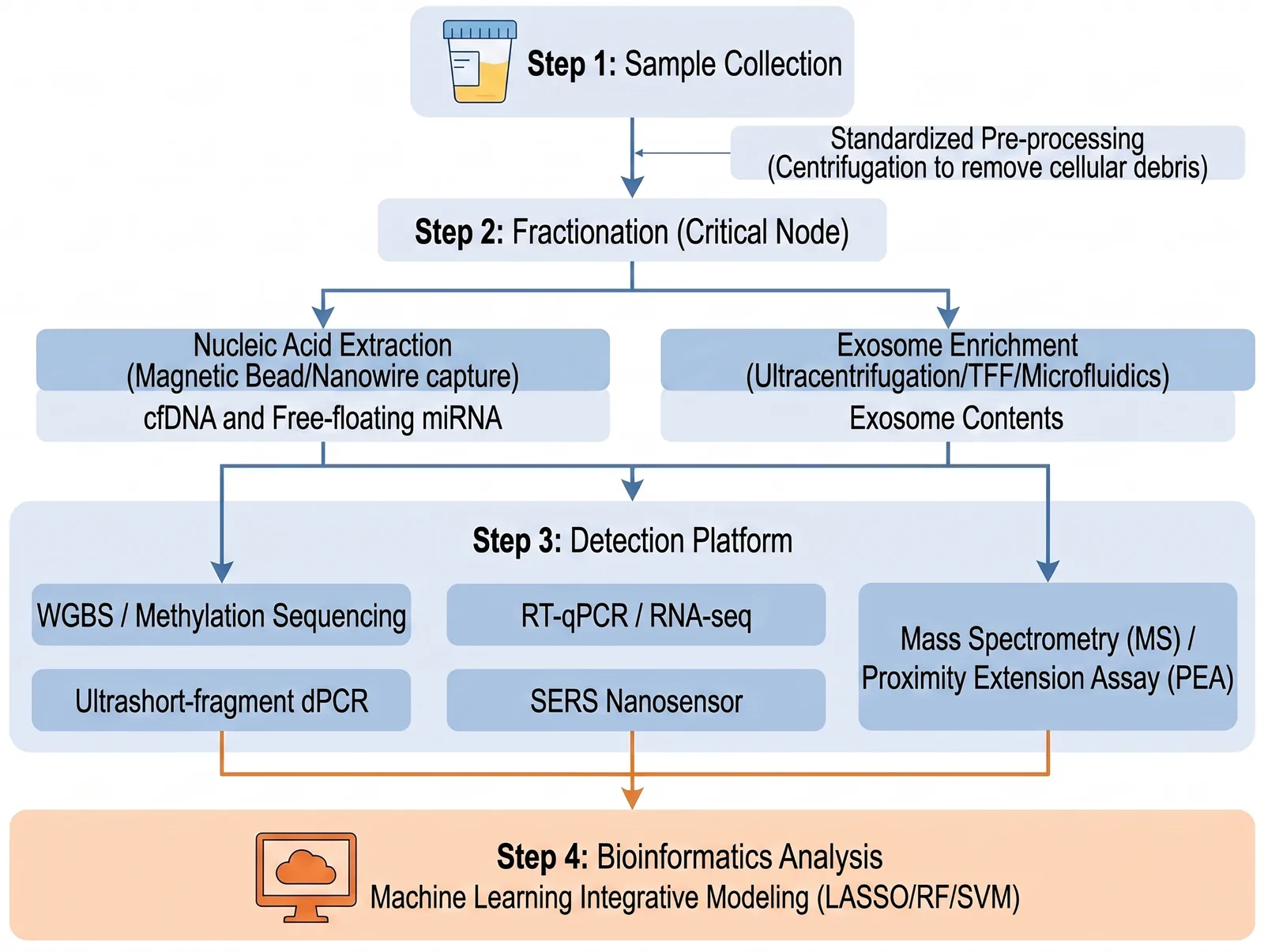
Technical workflow for urinary multi-omics biomarker detection and analysis. This flowchart illustrates the sequential steps from urine sample collection to data integration and clinical interpretation. The workflow begins with morning urine collection and standardized pre-analytical processing (centrifugation to remove cellular debris). The processed sample then branches into three parallel isolation pathways (1): nucleic acid extraction (magnetic bead-based or nanowire capture) for cfDNA and cell-free miRNA; (2) EV enrichment (ultracentrifugation, tangential flow filtration, or microfluidic platforms) for EV cargo analysis; and (3) direct supernatant analysis for protein and metabolite profiling. Downstream detection platforms include whole-genome bisulfite sequencing (WGBS) and ultra-short fragment digital PCR for cfDNA methylation/mutation analysis; RT-qPCR and RNA sequencing for miRNA expression profiling; surface-enhanced Raman scattering (SERS) for exosomal miRNA quantification; and mass spectrometry (MS) or proximity extension assay (PEA) for proteomic analysis. The final step integrates multi-omics data using machine learning algorithms (LASSO, RF, and SVM) to construct diagnostic or prognostic models. This workflow consolidates the diverse technical platforms discussed in Section 2 and underscores the analytical complexity underlying urinary biomarker development. cfDNA, cell-free DNA; LASSO, least absolute shrinkage and selection operator; miRNA, microRNA; MS, mass spectrometry; PEA, proximity extension assay; RF, random forest; RT-qPCR, reverse transcription quantitative polymerase chain reaction; SERS, surface-enhanced Raman scattering; SVM, support vector machine; TFF, tangential flow filtration; WGBS, whole-genome bisulfite sequencing.

### 2.1 Mechanisms of release and detection techniques for circulating free DNA and DNA methylation markers in urine

Urine is a rich source of cell-free DNA (cfDNA), which can originate from renal filtration of plasma cfDNA or direct shedding from the urogenital tract. In terms of fragmentome characteristics, renal-filtered urinary cfDNA is typically shorter and C-rich at the 5’ end. It is enriched in open chromatin regions and depleted in heterochromatic regions, indicating that the glomerular filtration barrier is selective regarding cfDNA size (12).

Furthermore, tumor-derived cell-free DNA fragments are primarily ultra-short (less than 50 bp) and are easily missed by conventional detection methods. Emerging techniques such as ultra-short-fragment digital PCR have shown promise in improving detection sensitivity in proof-of-concept studies. Still, they remain in early exploration stages and require further validation before clinical application (13).

For detection and methylation analysis, researchers have used whole-genome bisulfite sequencing (WGBS) and deep methylation sequencing to identify highly methylated biomarkers in urinary tumor DNA. To standardize data analysis, researchers have developed unified workflows such as cfDNA UniFlow, covering the entire process from raw sequencing data processing and error correction to copy number variation detection (14). In addition, zinc oxide nanowire-based capture technologies can efficiently enrich urinary cfDNA through hydrogen bonding and surface charge interactions, providing a new tool for molecular subtype identification (15).

### 2.2 Characterization of urinary microRNA expression profiles and quantitative analysis methods

MicroRNAs (miRNAs) play important functional and biomarker roles in malignant tumors of the urinary system. Their stable presence in urinary extracellular vesicles (EVs) eliminates the need for invasive procedures (16). Analysis of miRNA profiles in urinary EVs has shown that their expression patterns closely relate to the source-tissue cells and that different types of non-coding RNAs are subject to distinct sorting mechanisms (17). Studies have shown that miRNAs possess sorting sequences that determine whether they are secreted into small extracellular vesicles or retained intracellularly, and that specific RNA-binding proteins participate in the export process of miRNAs carrying specific motifs (18). RNA sequencing revealed that urinary EVs primarily express bladder-tissue-specific genes and are highly involved in immune activity, supporting their potential as reliable biomarkers in precision medicine (19). For quantitative analysis, researchers widely use traditional approaches such as RT-qPCR to validate the expression levels of specific miRNAs (e.g., miR-16, miR-21-5p, etc.) in urinary EVs, and in-house extraction kits can significantly shorten extraction time and reduce costs (20, 21). These studies were performed in non-small-cell lung cancer and prostate cancer, respectively, and are cited only to illustrate methodological advances in urinary exosomal RNA extraction and quantification, not as evidence for urothelial carcinoma. To overcome sensitivity limitations, researchers developed a surface-enhanced Raman scattering (SERS) sensing platform based on three-dimensional hierarchical plasmonic nanostructures for label-free, quantitative miRNA detection in urinary EVs. In a prostate cancer study, this platform achieved a detection limit of 10 aM for target miRNAs, a sensitivity improvement of more than 1,000-fold over previously reported methods, without complex pretreatment steps (22). This study is cited here as a technical proof of concept for ultrasensitive urinary EV-associated miRNA detection. Direct validation in urothelial carcinoma is still needed. Furthermore, free miRNA signatures in urine can support early tumor diagnosis, further expanding the scope of urine miRNA detection (23). This study is cited as a technical example of paired tumor–plasma–urine miRNA analysis rather than as direct evidence for urothelial carcinoma.

### 2.3 Urinary extracellular vesicles: isolation, characterization, and cargo analysis

Urinary extracellular vesicles are nanoscale (30–150 nm) membrane-bound vesicles of endosomal origin that carry a diverse cargo of proteins, lipids, and nucleic acids reflecting the physiological state of their cells of origin (24, 25). The term “exosome” specifically refers to a subset of EVs of endosomal origin. However, because most urinary isolation methods do not distinguish exosomes from other EV subtypes, this review uses the broader term “extracellular vesicles” unless endosomal origin is explicitly demonstrated. Their lipid bilayer resists enzymatic degradation, making them an attractive substrate for liquid biopsy (16, 26). However, clinical translation of exosome-based diagnostics depends critically on robust isolation and analytical technologies, which remain areas of active methodological development.

For EV isolation, differential ultracentrifugation remains the most widely used method, though it suffers from low purity and co-precipitation of non-exosomal contaminants. Tangential flow filtration (TFF) offers improved yield and scalability, while microfluidic technologies have enhanced isolation efficiency from small-volume samples, laying the foundation for point-of-care applications (27, 28). Immunoaffinity-based capture has been reported to provide high purity by targeting EV surface markers but is cost-prohibitive for routine use (28). For cargo characterization, proximity extension assay (PEA) and bead-based flow cytometry are employed to profile exosomal protein composition and surface protein expression (27). Immunogold-labeled transmission electron microscopy is used to confirm the spatial localization of DNA bound to EVs (29). The study was conducted in kidney allograft injury and is cited here only as a methodological proof of concept for detecting EV-bound DNA in urine. Its findings should not be extrapolated directly to urothelial carcinoma. At the transcriptomic level, RNA sequencing has enabled the discovery of EV-associated mRNAs and small nucleolar RNAs (snoRNAs) as potential cancer classifiers (30–32).

### 2.4 Translational challenges and standardization gaps

Despite their biological appeal, translating urinary EV-based diagnostics into clinical use faces substantial pre-analytical and analytical hurdles. The lack of standardized isolation protocols remains a critical bottleneck, as different methods yield EV populations with varying purity and integrity. Commercial precipitation-based kits offer convenience but introduce polymer contaminants that interfere with downstream analyses. The inherent heterogeneity of urinary EVs—derived from multiple cell types along the urogenital tract—makes it technically challenging to distinguish tumor-derived EVs from benign counterparts. Furthermore, the absence of universally accepted reference materials precludes cross-study comparability and hampers regulatory approval. The International Society for Extracellular Vesicles (ISEV) has published the MISEV2023 guidelines to address these issues, but adherence remains inconsistent across the field (33). Until the field systematically addresses these challenges, urinary EV biomarkers will remain predominantly research tools rather than clinically actionable diagnostic aids.

### 2.5 Emerging analytical platforms: urinary proteomics and metabolomics

This section provides a technical overview of urinary proteomics and metabolomics as emerging analytical platforms, emphasizing methodological capabilities and translational challenges. It is not intended as a systematic review of proteomic or metabolomic biomarkers in urothelial carcinoma. Where possible, we prioritize studies performed in urothelial carcinoma or bladder cancer; evidence from other diseases or tumor types is identified as such and is cited only to illustrate technical feasibility or methodological advances. Urine contains a variety of metabolites and proteins. Advances in multi-omics technologies, such as proteomics and metabolomics, have improved the sensitivity and specificity of urinary tract cancer analysis, thereby enhancing diagnostic accuracy (3). In proteomics, urinary EV protein profiles differ significantly in patients with urothelial carcinoma. Studies have identified unique protein signatures associated with muscularis invasion, with proteins such as HO-1 and MMP7 confirmed as potential non-invasive prognostic tools (27). In addition to EV proteins, urine chemokines and nucleosomes are also considered potential non-invasive biomarkers for assessing urological pathology and long-term prognosis (34, 35). At the metabolomics level, metabolites contained in EVs can exert biological functions and serve as surrogate biomarkers reflecting the metabolic state of tumor cells (16, 24, 25). Some of these studies were conducted in non-urothelial carcinoma settings and are cited only to illustrate analytical feasibility; their findings should not be extrapolated directly to urothelial carcinoma. In urothelial carcinoma, urinary metabolomic profiling has identified alterations in tryptophan, carnitine, and phospholipid metabolism, as well as changes in polyamine and bile acid pathways, some of which correlate with tumor stage and grade (36, 37). However, most metabolomic studies remain exploratory, with small sample sizes, limited external validation, and inconsistent metabolite annotation across platforms. Moreover, the overlap between tumor-derived and host-derived metabolites in urine complicates the interpretation of metabolomic signals. These limitations currently restrict the clinical utility of urinary metabolomics in urothelial carcinoma. Given the limitations of single-omics approaches, current liquid biopsy platforms are shifting from single markers to composite biomarker panels that integrate multi-omics information; this shift relies heavily on advanced computational and analytical methods (38). In urothelial carcinoma, urinary metabolomics remains less mature than genomics, epigenomics, or proteomics, and its incremental value within multi-omics models remains unestablished.

These technological platforms—ranging from cfDNA capture and methylation sequencing to exosome isolation and proteomic profiling—provide the analytical foundation upon which the clinical performance of urinary biomarkers depends. Translating these technical capabilities into clinically meaningful diagnostic tools depends critically on robust clinical validation, as discussed in the following section. In the following section, we critically evaluate the reported sensitivity and specificity of each biomarker class in early screening and non-invasive diagnosis, paying particular attention to the crucial distinction between proof-of-concept accuracy in carefully selected cohorts and the performance that is realistically expected in unselected, real-world screening populations. As the guidelines discussion in Section 3.5 will underscore, the primary literature often overlooks this distinction (**Figure 2**). In the following section, we evaluate clinical performance specifically in urothelial carcinoma. We do not use evidence from other tumor types to estimate sensitivity or specificity in urothelial carcinoma. We use it only to illustrate technical feasibility or methodological advances.

## 3 Evaluation of the efficacy of urinary multi-omics biomarkers in early screening and non-invasive diagnosis

Having established the technical platforms that enable urinary multi-omics detection in Section 2, we now critically evaluate their clinical performance. Rather than presenting a catalog of biomarkers organized by molecular type, we structure this section around the three clinical scenarios where urinary biomarkers are most needed: (1) early detection and screening; (2) staging and grade discrimination; and (3) postoperative monitoring and recurrence prediction. For each scenario, we compare the performance of different biomarker classes and, crucially, identify the evidence gaps that limit clinical adoption. This problem-driven organization reflects the central argument of this review: that the value of urinary biomarkers must be assessed not by per-analyte performance in isolation, but by their ability to address specific clinical needs. The studies reviewed in this section were conducted in clinically heterogeneous populations, including patients with bladder urothelial carcinoma, upper tract urothelial carcinoma (UTUC), non-muscle-invasive bladder cancer (NMIBC), muscle-invasive bladder cancer (MIBC), hematuria, and postoperative surveillance cohorts. These settings differ substantially in disease prevalence, pre-test probability, diagnostic gold standard, and clinical endpoint. Diagnostic performance estimates are therefore not directly comparable across studies unless the tumor type, disease stage, and clinical scenario are matched. Throughout this section, we specify the tumor type (bladder urothelial carcinoma vs. UTUC) and clinical setting (screening, symptomatic diagnosis, hematuria triage, staging, or surveillance) wherever the original study permits. Comparisons across biomarker classes are presented within, rather than across, these clinical contexts.

### 3.1 Sensitivity and specificity of DNA methylation and cfDNA mutations in early detection

Most evidence in this subsection comes from patients with bladder urothelial carcinoma, with one notable prospective cohort in UTUC. Bladder urothelial carcinoma and UTUC are distinct clinical entities with different risk profiles and diagnostic pathways, and their performance estimates should not be directly compared. The primary challenge in early detection lies in identifying low-grade and small-volume tumors, for which urinary cytology has notoriously low sensitivity. Several urinary multi-analyte assays have shown promising diagnostic performance in the specific early detection or diagnostic cohorts in which they were evaluated. DNA methylation markers, which are frequently altered in the early stages of carcinogenesis, have shown diagnostic utility in several prospective cohorts. A prospective multicenter study demonstrated that quantitative detection of H4C6 and TWIST1 gene methylation in morning urine achieved a diagnostic sensitivity of 93.02% (95% CI 90.01–95.35%) and a specificity of 92.13% (95% CI 88.83–94.71%) for urothelial carcinoma (39). Separately, the UriMee model—developed using a model cohort (n = 322) and validated in an independent cohort (n = 131)—achieved 92% sensitivity and 92% specificity for UC detection, with 96% sensitivity for UTUC (40). It significantly outperformed urine cytology (90% vs. 25% sensitivity) in the same study (40). These two assays are methodologically distinct: H4C6/TWIST1 is a two-gene methylation panel evaluated in a prospective multicenter diagnostic accuracy study, whereas UriMee is a multi-marker methylation model developed and validated in separate model and validation cohorts. For UTUC, the Bladder CARE test, based on methylation levels of TRNA-Cys, SIM2, and NKX1-1, achieved 96% sensitivity and 88% specificity in a prospective UTUC cohort (41). In this UTUC setting, it substantially outperformed traditional cytology, which had a 63% false-negative rate. This result should not be extrapolated directly to bladder urothelial carcinoma screening or surveillance. Beyond methylation, integrated panels combining multiple analytes have also shown promising performance in the cohorts studied. The multi-target urinary tumor DNA detection method UI-Seek integrates FGFR3 and TERT mutation data with ONECUT2 and VIM methylation data, achieving an overall sensitivity of 91.37% and a specificity of 95.09%. Critically, its sensitivity reached 75.81% for low-grade Ta-stage tumors, a demographic where cytology is essentially ineffective (42). These findings suggest that multi-analyte panels—including multi-marker methylation assays and integrated genomic–epigenomic tests—may improve non-invasive detection in the specific populations and clinical settings studied, particularly for low-grade Ta bladder tumors and for UTUC in a prospective cohort. However, these estimates derive from heterogeneous studies with different tumor types (bladder urothelial carcinoma vs. UTUC), clinical scenarios (symptomatic diagnosis vs. early detection), and sample sizes. Because external validation remains limited, they should not be generalized to unselected screening populations or to all patients with urothelial carcinoma. Whether these panels can substantially improve early detection in routine practice requires further prospective, multicenter validation in clinically representative cohorts. These findings should therefore be regarded as hypothesis-generating and context-dependent rather than as evidence that multi-analyte panels are broadly effective for early screening.

### 3.2 The diagnostic value of urinary miRNA profiles in distinguishing NMIBC from MIBC in bladder urothelial carcinoma

This subsection focuses on preoperative staging in bladder cancer, specifically the discrimination between NMIBC and MIBC. The cited studies were conducted in bladder cancer cohorts. Their applicability to UTUC staging remains unclear. The distinction between non-muscle-invasive bladder cancer (NMIBC) and muscle-invasive bladder cancer (MIBC) is a critical determinant of treatment strategy and prognosis. Yet, current non-invasive tools provide limited guidance for preoperative staging. Urinary microRNAs (miRNAs), given their role in regulating epithelial-mesenchymal transition, invasion, and tumor progression, have been increasingly investigated for their potential to stratify patients by invasion depth (43).

Multiple independent cohorts have provided preliminary evidence suggesting that urinary miRNAs may differ between NMIBC and MIBC. In a study using high-throughput sequencing of urinary exosome-derived miRNAs followed by RT-qPCR validation in 53 bladder cancer patients, miR-93-5p was significantly elevated in MIBC compared with NMIBC, with an area under the ROC curve (AUC) of 0.769 for distinguishing the two groups (44). Urinary exosomal miR-93-5p expression levels also correlated with TNM stage (Ta-T1 vs T2-T4), consistent with a possible association with invasion; however, given the small sample size (N = 53) and single-center design, this finding should be regarded as hypothesis-generating rather than as evidence supporting clinical staging utility. In the same study, researchers identified 61 differentially expressed miRNAs between MIBC and NMIBC, underscoring the molecular heterogeneity captured by urinary exosomal profiling.

Beyond exosomal miRNAs, cell-free urinary miRNA panels have also demonstrated stage-discriminatory capacity. A seven-miRNA panel (miR-7-5p, miR-22-3p, miR-29a-3p, miR-126-5p, miR-200a-3p, miR-375, and miR-423-5p) achieved AUCs of 0.864, 0.930, and 0.978 for Ta, T1, and T2-T4 tumors, respectively, significantly outperforming urine cytology (45). This gradient of increasing AUC with advancing stage suggests that urinary miRNA signatures may be particularly informative for detecting muscle invasion. Researchers have also investigated specific individual miRNAs. A systematic review of urinary miRNAs in bladder cancer summarized evidence for markers such as miR-145, miR-205, and miR-182. However, because of substantial heterogeneity in study design, sample source, normalization strategies, and endpoint definitions, the review did not provide a uniform pooled estimate for stage discrimination (46). These markers should therefore be regarded as exploratory rather than clinically validated staging biomarkers (46). In urinary extracellular vesicles, miR-146b-5p (P = 0.004) and miR-155-5p (P = 0.036) exhibited significantly higher expression in MIBC patients (47). A recent 2025 study utilizing next-generation sequencing of urinary small RNAs in 52 treatment-naïve patients identified 1,967 differentially expressed miRNAs between MIUC and NMIUC, with hsa-miR-3168 showing the most pronounced upregulation (10.2-fold) in muscle-invasive disease (48).

These accumulating data suggest that urinary miRNA profiles, whether derived from exosomes or cell-free fractions, hold considerable potential for non-invasive staging of urothelial carcinoma. However, several limitations warrant acknowledgment. First, most published studies have modest sample sizes, and the reported AUCs—while promising—have not been consistently validated in large, independent cohorts. Second, the optimal miRNA panel and the choice between exosomal and cell-free sources remain unresolved, with different studies using different platforms and normalization strategies. Third, the clinical utility of miRNA-based staging will ultimately depend on its performance relative to, or in combination with, imaging and other liquid biopsy modalities. Prospective, multicenter studies with standardized pre-analytical protocols are urgently needed to validate these findings and establish whether urinary miRNA signatures can reliably inform clinical decisions about the need for radical versus bladder-preserving therapy. Moreover, as reflected in **Table 1**, several early-stage miRNA studies report AUCs without corresponding sensitivity or specificity values, limiting comprehensive performance comparisons across marker classes. This reporting gap underscores the preliminary, exploratory nature of many urinary miRNA investigations. It highlights the need for future studies to follow standardized reporting guidelines, including full disclosure of diagnostic performance metrics, to enable meaningful cross-study comparisons and meta-analysis.

**Table 1:** Performance summary of key urinary biomarker panels for urothelial carcinoma.

Biomarker/panel	Sample type	Tumor type	Omics layer	Clinical setting/endpoint	Study design (N)	Key performance	Ref.
H4C6 + TWIST1 methylation	Morning urine	UC	DNA methylation	UC detection	Prospective, multicenter diagnostic accuracy study (N = 743 urine samples)	Sensitivity 93.02% (95% CI 90.01–95.35); specificity 92.13% (95% CI 88.83–94.71)	(38)
UriMee	Urine	UC (bladder and UTUC)	DNA methylation	UC early diagnosis; UTUC subgroup	Model cohort (n = 322); independent validation cohort (n = 131)	Sensitivity 92%; specificity 92%; UTUC sensitivity 96%; urine cytology sensitivity 25% in the same study	(39)
Bladder CARE (TRNA-Cys, SIM2, NKX1-1)	Urine	UTUC	DNA methylation	UTUC detection	Prospective UTUC cohort (N = 50; 40 radical nephroureterectomy, 7 ureterectomy, 3 ureteroscopy)	Sensitivity 96%; specificity 88%; cytology false-negative rate 63%	(40)
UI-Seek (FGFR3, TERT mutations + ONECUT2, VIM methylation)	Urine	UC (bladder and UTUC)	Genomics + epigenomics	UC detection, including low-grade Ta	Retrospective model cohort (n = 382); prospective, double-blinded, multicenter validation (n = 947)	Overall sensitivity 91.37%; specificity 95.09%; low-grade Ta sensitivity 75.81%; high-grade sensitivity >93%	(41)
VIM + TMEM220 + PPM1N methylation	Urine	UC (NOS)	DNA methylation	UC early detection	Validation cohort (N = 432; 108 UC, 191 non-neoplastic individuals, 76 benign lesions, 57 other cancers)	Overall sensitivity 94.44%; specificity 93.52%; stage 0a/0is sensitivity 80%; stage I sensitivity 88.57%	(75)
PENK methylation	Urine	Bladder cancer (BC)	DNA methylation	BC diagnosis	Prospective multicenter study at 10 sites in the Republic of Korea (N = 1099; 219 BC, 176 high-grade or invasive BC)	Sensitivity 89.2% for high-grade/invasive BC; 78.1% for all stages/grades; reported higher than NMP22/cytology within the same study; indirect comparison	(50)
FGFR3/TERT/HRAS mutations + OTX1/ONECUT2/TWIST1 methylation	Urine	Hematuria (risk stratification for UC)	Genomics + epigenomics	Hematuria risk stratification	Prospective Dutch hematuria cohort with complete biomarker status (n = 838; low risk 65, intermediate 106, high 667)	AUC 0.929–0.971 across hematuria risk groups	(74)
CXCL16 + TGFBI + DNA methylation markers	Urine	UC (NOS)	Proteomics + epigenomics	UC detection among genitourinary diseases and cancer-free individuals	Independent cross-sectional study (N = 1119 urine samples)	Sensitivity 72%; specificity 97%	(69)
Urinary EV proteome classifier	Urinary EVs	Bladder cancer (BC)	Proteomics	BC diagnosis; muscle invasiveness	BC patients (N = 39) vs healthy controls, prostate cancer patients, and whole urine	Accuracy 92%; associated with muscle invasiveness	(27)
7-miRNA panel (miR-7-5p, miR-22-3p, miR-29a-3p, miR-126-5p, miR-200a-3p, miR-375, miR-423-5p)	Urine (cell-free)	Bladder cancer (BC)	miRNA	BC diagnosis and stage discrimination	276 BC patients and 276 controls; genome-wide deep sequencing followed by two-phase RT-qPCR; training and validation sets	AUC 0.864 (Ta), 0.930 (T1), 0.978 (T2–T4); outperformed cytology	(44)
EV-associated miR-93-5p	Urinary EVs	Bladder UC (NMIBC vs MIBC)	miRNA	MIBC vs NMIBC	High-throughput sequencing + RT-qPCR validation (N = 53)	AUC 0.769; correlated with TNM stage	(43)
miR-146b-5p and miR-155-5p	Urinary extracellular vesicles	Bladder UC (NMIBC vs MIBC)	miRNA	MIBC vs NMIBC	Microarray analysis in 24 FFPE tumor samples; qRT-PCR validation in 56 FFPE tumor samples and 37 urinary EV samples	Higher expression in MIBC (P = 0.004 and P = 0.036, respectively)	(46)
hsa-miR-3168	Urine	UC (NOS; muscle-invasive vs non-muscle-invasive)	Small RNA	Muscle-invasive vs non-muscle-invasive UC	NGS of urinary small RNAs in treatment-naive patients (N = 52)	1,967 differentially expressed miRNAs; hsa-miR-3168 upregulated 10.2-fold in muscle-invasive disease	(47)
Urinary EV-associated mRNAs SRGN, FLI1, MACROH2A2	Urinary EVs	Bladder cancer (BC)	mRNA/transcriptomics	Early-stage BC vs healthy controls	Transcriptomic sequencing of normal and BC cell lines; RT-qPCR profiling in clinical urine samples (n = 97: healthy controls, BC patients, post-surgery BC patients)	BC diagnosis AUC 0.973; early-stage BC vs healthy controls AUC 0.969; downregulated postoperatively.	(32)

Data are summarized from the cited original studies, except where a systematic review is explicitly indicated.

Setting indicates the clinical context in which the assay was evaluated. Design (N) summarizes the study design and sample size. Sensitivity, specificity, and AUC are reported as percentages or proportions; 95% CIs are shown where available. Because assays were evaluated in different tumor types (bladder UC vs UTUC), clinical settings (screening, symptomatic diagnosis, hematuria triage, staging), disease spectra, and reference standards, performance estimates should not be compared directly across rows. Non-urinary and non-urothelial carcinoma evidence is not included.

AUC, area under the receiver operating characteristic curve; BC, bladder cancer; CI, confidence interval; EV, extracellular vesicle; MIBC, muscle-invasive bladder cancer; miRNA, microRNA; N, number; NMIBC, non-muscle-invasive bladder cancer; NMP22, nuclear matrix protein 22; NR, not reported; PENK, proenkephalin; PPM1N, protein phosphatase Mg2+/Mn2+ dependent 1N; RT-qPCR: reverse transcription quantitative polymerase chain reaction; TMEM220, transmembrane protein 220; UC, urothelial carcinoma; UTUC, upper tract urothelial carcinoma; VIM, vimentin.

### 3.3 Diagnostic performance of urinary EV-associated markers in low-grade and early-stage bladder urothelial carcinoma

This subsection focuses primarily on bladder urothelial carcinoma, particularly low-grade and early-stage lesions. Some platform-level studies were performed in prostate cancer and are cited only as technical examples; they do not provide clinical performance estimates in urothelial carcinoma. Urinary EVs have attracted considerable interest as diagnostic substrates, particularly for low-grade and early-stage urothelial carcinoma, where conventional cytology performs poorly. Advances in sensitive detection platforms have improved the analytical sensitivity of urinary EV-associated miRNA detection. However, most such platforms have not yet been validated for clinical performance in urothelial carcinoma. For example, a three-dimensional nanomachine based on a Janus wireframe DNA cube enables rapid fluorescent detection of exosomal miRNA-21 through catalytic hairpin assembly, with a picomolar detection limit (49). At the transcriptomic level, a study identified three mRNAs—SRGN, FLI1, and MACROH2A2—in urinary extracellular vesicles (EVs) as non-invasive diagnostic markers for bladder cancer through transcriptomic sequencing. The reported AUC was 0.973 for bladder cancer diagnosis and 0.969 for distinguishing early-stage bladder cancer patients from healthy controls. These markers were significantly downregulated in urine samples from postoperative patients, suggesting their origin from tumor tissue (32). EV-associated circular RNAs are also dysregulated in urinary tract tumors, demonstrating strong potential as targets for early diagnosis (50). While these findings are encouraging, a critical question remains: do EV-associated markers offer any tangible advantage over their non-EV-associated counterparts, such as cell-free DNA (cfDNA) or free-floating miRNAs in urine? The literature often asserts that EV-associated markers are “more sensitive” or “more stable,” but these claims warrant closer scrutiny.

#### 3.3.1 EV-associated vs. non-EV-associated analytes: a comparative perspective

A balanced evaluation of exosomal versus non-exosomal urinary biomarkers requires examining their biological and practical attributes across multiple dimensions. Biological advantages of EV-associated markers. Mechanistically, EV cargo is actively sorted and encapsulated within a lipid bilayer, conferring intrinsic protection against RNases and other degradative enzymes in urine. This contrasts with free-floating miRNAs, which are exposed to the harsh urinary environment and undergo substantial degradation during storage or freeze-thaw cycles. Furthermore, EV-associated miRNA and protein profiles are not merely passive waste products but reflect the active biological state of their source cells, including tumor-specific sorting mechanisms that may enrich certain oncogenic transcripts (17, 18). This active sorting could provide a biological enrichment step that theoretically improves the signal-to-noise ratio for tumor-derived transcripts, particularly low-abundance targets that might be obscured in the total urinary miRNA pool.

Technical and practical limitations. However, these theoretical advantages are substantially offset by practical limitations. First, separating and purifying exosomes from urine remains technically demanding, with significant inter-laboratory variability and poor scalability for high-throughput clinical testing. In contrast, cfDNA extraction from urine is relatively straightforward, well-standardized, and compatible with automated platforms. Second, tumor-derived exosomes represent only a minor fraction of the total exosomal population in urine, which is dominated by vesicles derived from normal urothelial, renal, and inflammatory cells. This low abundance in early-stage disease fundamentally limits the sensitivity of exosome-based approaches, irrespective of the sophistication of the detection platform. Third, while EV cargoes are protected from enzymatic degradation, they are not protected from loss during isolation, and current exosomal RNA recovery rates remain suboptimal for many clinical applications.

Implications for clinical application scenarios. These comparative considerations suggest that exosomal markers and non-exosomal markers are not interchangeable but rather suited to different clinical contexts. For primary screening and early diagnosis, where simplicity, scalability, and cost-effectiveness are paramount, cfDNA methylation panels currently hold a distinct advantage due to more mature workflows and robust performance in large cohorts. For post-operative minimal residual disease (MRD) monitoring, exosomal miRNA may offer a unique advantage. The rationale is twofold: (1) the active sorting mechanism may enrich transcripts associated with residual or proliferating tumor cells, potentially providing an earlier signal of recurrence than passively shed cfDNA; and (2) the higher stability of exosomal RNA permits more reliable detection in serial samples subjected to variable pre-analytical conditions. Nevertheless, this hypothesis remains to be prospectively validated, and head-to-head comparisons of exosomal and non-exosomal markers in the same patient cohorts are conspicuously absent from the literature. For now, interpret the superior sensitivity of exosomal platforms in research settings with caution, as it has not translated into a clear, reproducible clinical advantage over cfDNA-based approaches in real-world studies. Critically, direct head-to-head comparisons of exosomal and non-exosomal markers in the same patient cohorts remain conspicuously absent from the literature. Consequently, the widely cited assertion that exosomal markers are inherently “superior” or “more stable” remains a hypothesis requiring prospective validation, rather than an established conclusion. Until such comparative data emerges, both EV-associated and non-EV-associated approaches should be viewed as complementary strategies, each with distinct strengths and limitations, rather than one being categorically superior to the other.

### 3.4 Comparison of the efficacy of multi-omics biomarkers with conventional urine cytology and cystoscopy

The comparative studies in this subsection span different clinical settings, including symptomatic diagnosis, hematuria risk stratification, and postoperative surveillance, and include both bladder urothelial carcinoma and UTUC. We therefore specify the setting and tumor type for each assay rather than pooling performance estimates. Although traditional urinary cytology has high specificity, its sensitivity for low-grade and early-stage urothelial carcinoma is generally low, making it difficult to meet the clinical needs of early screening. In the studies reviewed, urinary multi-analyte biomarker assays have shown promising diagnostic performance. Importantly, the nature of the comparison with conventional tests varies across studies. Some assays have been compared with cytology, NMP22, or UroVysion FISH within the same study, whereas other comparisons remain indirect because they derive from different cohorts. In the same study, the UriMee DNA methylation model achieved 92% sensitivity, compared with 25% for urinary cytology, while maintaining comparable specificity (40). PENK methylation testing achieved a sensitivity of 89.2% for high-grade or invasive bladder cancer and 78.1% for bladder cancer across all stages and grades. Within the same prospective multicenter study, these estimates were higher than those reported for NMP22 testing or urine cytology (51). However, this within-study comparison does not replace independent external head-to-head validation.

Comparisons between multi-analyte panels and conventional tests in this subsection are therefore not uniformly indirect. Some, including PENK methylation and UI-Seek, were performed within the same prospective study, whereas others, such as UriMee versus FISH, are indirect. Even when a direct within-study comparison is available, it derives from a single trial and requires independent external validation. Head-to-head comparative studies across independent cohorts are still needed to determine relative diagnostic performance.

In its own prospective, double-blinded, multicenter trial, the multi-target UI-Seek assay showed high sensitivity and specificity. In that same trial, UI-Seek showed improved sensitivity over urine cytology, the NMP22 test, and UroVysion FISH, with comparable specificity (42). In the original study, orthogonal Sanger sequencing supported the analytical validity of the single-target assay (42). However, these results come from one trial and require independent external validation before they can be considered definitive. Preliminary data reported in a research letter suggested that a urine DNA assay (utLIFE-UC) may have higher sensitivity than cytology in the studied cohort, including detection of residual disease. However, because this evidence was reported as a letter rather than a full-length original clinical study, it is not used here to estimate diagnostic performance and requires prospective validation (52).

Beyond cytology, several urine-based tests have received FDA clearance for bladder cancer surveillance, most notably NMP22 (a nuclear matrix protein immunoassay) and UroVysion FISH (53). However, their clinical adoption has been limited by intrinsic performance constraints. NMP22 has moderate sensitivity and high false-positive rates due to benign inflammatory conditions and hematuria, which severely compromise its specificity in real-world practice. UroVysion FISH offers superior specificity and better sensitivity for high-grade lesions. Still, its sensitivity for low-grade Ta tumors remains disappointingly low (approximately 30–40%), precisely the demographic that most needs non-invasive testing. Moreover, FISH is costly, requires specialized equipment and experienced cytotechnologists for interpretation, and has low throughput, limiting its utility for large-scale screening or frequent surveillance.

In this context, the next-generation multi-analyte panels reviewed here have shown promising performance in their respective studies. These comparisons should still be interpreted with caution because most are derived from individual studies, and independent external validation remains limited. In the UI-Seek trial, the assay showed higher sensitivity than cytology, NMP22, and UroVysion FISH, and achieved 75.81% sensitivity for low-grade Ta tumors (42). UriMee reached 92% overall sensitivity (40); for UriMee, no head-to-head comparison with FISH has been made. Whether these panels are truly more accessible or cost-effective than existing tests requires prospective, head-to-head comparative studies with health-economic evaluation. Second, by combining methylation, mutation, and protein signatures, multi-analyte models may reduce the false-positive burden associated with inflammation that limits NMP22, although this has not been directly compared. Third, shallow-depth sequencing approaches such as UCseek maintain diagnostic performance at ultra-low sequencing depths in their derivation cohorts, which may reduce per-test costs (54). However, formal cost-effectiveness analyses are lacking. While FISH remains a valuable adjunct in specific scenarios, particularly for adjudicating equivocal cytology in high-risk patients, multi-analyte panels may offer greater flexibility and scalability. Whether they are truly more accessible or cost-effective than existing tests requires prospective, head-to-head comparative studies with health-economic evaluation.

In clinical practice, traditional urinary cytology has a high false-negative rate. In a UTUC cohort, the urine-based epigenetic Bladder CARE test identified most patients, whereas cytology had a 63% false-negative rate (41). This finding is specific to UTUC and should not be generalized to bladder urothelial carcinoma surveillance. Although cystoscopy remains the gold standard for diagnosis and a cornerstone of NMIBC surveillance, its invasiveness, cost, and patient discomfort limit its use for large-scale population screening and may make less frequent surveillance desirable in selected low-risk patients. Because of their non-invasive nature and promising diagnostic performance, urinary multi-omics biomarkers may address some shortcomings of traditional urinary cytology. However, current evidence does not support reducing reliance on cystoscopy, as false-negative results from urine tests could lead to delayed diagnosis of high-grade or muscle-invasive disease. At present, these tests are best viewed as supplementary tools that may guide the more selective use of cystoscopy in specific clinical scenarios, rather than as replacements for the gold-standard examination. However, this pragmatic approach should not be misinterpreted as a denial of these studies’ potential. It reflects the current level of evidence and provides a starting point for the translational medicine roadmap outlined in Section 6.

### 3.5 Concluding remarks on clinical performance

In summary, the evidence reviewed in this section demonstrates that urinary multi-omics biomarkers have evolved from exploratory tools to potentially useful diagnostic adjuncts. In the studies reviewed, they have shown higher sensitivity than urinary cytology, have demonstrated complementary value to cystoscopy, and may offer advantages in early detection and postoperative surveillance (**Table 1**). Integrated models combining methylation, mutation, and protein signatures have achieved diagnostic accuracy exceeding 90% in prospective cohorts, addressing many historical limitations of non-invasive urine testing. However, a critical observation emerges from this evidence synthesis: despite the impressive performance figures, none of these tests have been widely integrated into routine clinical practice. This dissonance between research promise and clinical reality reflects deep-seated barriers spanning evidence quality, regulatory approval, cost-effectiveness, and healthcare system readiness—barriers that we systematically dissect in the following section. However, these estimates derive from different tumor types and clinical settings. Because bladder urothelial carcinoma, UTUC, hematuria triage, and surveillance cohorts differ in pre-test probability and endpoint, their reported performance should not be interpreted as directly comparable.

## 4 The application of urinary multi-omics biomarkers in postoperative longitudinal monitoring, recurrence prediction, and personalized follow-up management

The recurrence rate after surgery for urothelial carcinoma remains high, and establishing a long-term, dynamic monitoring system is important for improving patients’ long-term survival. The development of specific biomarker panels for recurrence monitoring has become an active area of clinical investigation.

### 4.1 Clinical significance and implementation strategies for longitudinal monitoring of postoperative urinary multi-omics biomarkers

Urothelial carcinoma has a high rate of postoperative recurrence, and long-term follow-up plays a critical role in improving patient prognosis. The 2022 European Association of Urology guidelines emphasize that recurrence cases detected during follow-up have a significantly better prognosis than symptomatic recurrence cases, highlighting the clinical value of rigorous postoperative monitoring (55).

Urine contains metabolites, proteins, shed cells, and cell-free DNA; its non-invasive nature and ease of collection make it an ideal liquid biopsy sample (3). Multi-omics technologies have significantly enhanced the sensitivity and specificity of analysis, improving disease-monitoring outcomes (3). Liquid biopsies, such as urinary tumor DNA, have been proven effective for detecting minimal residual disease (MRD) and for postoperative monitoring. They may help address the limitations of traditional tissue sampling, including limited availability and the inability to repeatedly capture tumor molecular heterogeneity over time, thereby advancing personalized patient monitoring (56).

In terms of implementation strategies, developing biomarker panels specifically targeted for recurrence monitoring is a current priority. For example, in a prospective cohort study involving 478 patients under follow-up, the BC-106 biomarker panel—analyzed using capillary electrophoresis-mass spectrometry—combined with cytology achieved an area under the receiver operating characteristic curve (AUC) of 0.82, significantly outperforming the 0.72 AUC of cytology alone (57). This combined model has a negative predictive value (NPV) exceeding 95% and may significantly reduce unnecessary cystoscopies during follow-up in clinical practice, thereby optimizing monitoring and alleviating the burden on patients (57). Furthermore, the nanomics paradigm—which combines multi-omics liquid biopsy with nanotechnology tools to enrich circulating tumor omics components—also provides a new technical approach for postoperative longitudinal monitoring (8).

### 4.2 Association between changes in urinary biomarker levels and tumor recurrence, progression, and muscle invasion

This subsection evaluates urinary biomarker evidence linking longitudinal changes in urinary multi-omics markers to tumor recurrence, progression, and muscle invasion. To provide mechanistic context, we also cite selected tissue-derived or systemic non-urinary studies. We explicitly label these as tissue-derived, non-urinary, or contextual evidence and do not use them to estimate the diagnostic or prognostic performance of urinary biomarkers. Clinical risk models and multimodal integrative frameworks are discussed in Section 4.3 and are not used here as urinary biomarker evidence. Longitudinal changes in urinary multi-omics biomarkers are closely associated with tumor recurrence, progression, and the extent of muscle invasion (**Table 2**). Regarding the omics characteristics of recurrence mechanisms, a multi-omics analysis of bladder cancer patients who underwent surgical treatment revealed that, in recurrent bladder cancer, 5hmC-associated transcriptional changes were significantly enriched in genes related to fatty acid oxidation. Concurrently, the study identified a series of 5mC hypomethylated differentially methylated regions in the NFATC1 genomic region; this gene is closely associated with T-cell immune responses (58). The study also noted that because 5mC and 5hmC alterations are globally negatively correlated, markers based on bisulfite-based methods that combine both may attenuate cancer-associated signals, thereby affecting their accuracy as clinical biomarkers for predicting recurrence (58). Regarding the association between specific mutations and recurrence, sequencing studies of flat urothelial lesions showed that KRAS G12C and ERBB2 S310F/Y mutations were detected only in carcinoma *in situ* and were significantly associated with recurrence following BCG therapy (59). Regarding tumor progression and muscle-invasive bladder cancer, a gain of chromosome 5p has been identified as a high-risk factor in non-muscle-invasive bladder cancer, as it promotes tumor invasion by regulating the actin cytoskeleton (60). For muscle-invasive urothelial carcinoma, high-throughput proteomics has identified TUBB6 as a potential biomarker for predicting invasiveness and poor prognosis, with its expression levels significantly correlated with overall survival (61). Furthermore, when assessing progression to muscle-invasive or higher stages, urinary DNA methylation panels—such as GDF15, TMEFF2, and VIM—have shown very high performance in predicting high-grade and ≥pT2 disease, with 91% sensitivity and 100% specificity (62). Systemic (blood-based) ctDNA levels have also been shown to increase with disease progression, suggesting that a higher tumor burden is associated with greater aggressiveness (63). This is a non-urinary analyte and is cited here only for comparison; Sections 4.1 and 4.3 discuss urinary tumor DNA separately.

**Table 2:** Summary of urinary multi-omics biomarker studies in postoperative recurrence monitoring for urothelial carcinoma.

Biomarker/panel	Sample type	Omics layer	Clinical setting/endpoint	Study design (N)	Key performance	Ref.
BC-106 + cytology	Urine	Proteomics/peptidomics (CE-MS)	Postoperative follow-up; recurrence monitoring	Prospective multicenter validation cohort (N = 478)	AUC 0.82 for BC-106 + cytology vs 0.72 for cytology alone; NPV >95%	(55)
UCseek	Urine sediment	Genomics + epigenomics	Recurrence monitoring	Discovery cohort: n = 167 (90 tumors, 77 without tumors); independent validation cohort from three hospitals: n = 206; recurrence/surveillance cohort: n = 42	Accuracy 90.91% vs cystoscopy 59.09% in recurrence monitoring cohort; maintained performance at ultra-low sequencing depth in derivation cohort	(52)
Urine cfDNA multi-omics (MAF, inferred TMB, copy-number-based tumor fraction)	Urine cfDNA	Genomics (cfDNA multi-feature: MAF, TMB, CNV)	MRD detection and survival prediction after treatment	Localized bladder cancer patients (N = 74)	Urine cfDNA VAF, inferred TMB, and CNV-derived tumor fraction predicted pCR better than plasma ctDNA; RF model (LOOCV) achieved 87% sensitivity for residual disease; predicted MRD correlated with worse PFS (HR = 3.00, p = 0.01) and OS (HR = 4.81, p = 0.009)	(72)

Data are summarized from the cited original studies. This table includes urinary molecular biomarker studies in postoperative recurrence monitoring or MRD detection for urothelial carcinoma; not all entries are strictly multi-omics. This table excludes non-urinary evidence, tissue-based molecular subtyping, blood ctDNA, clinical risk models, and multimodal imaging/pathology frameworks. Sample type, clinical endpoint, study design, and sample size are indicated where available. Because studies differ in sample type, disease stage, treatment history, and endpoint, do not compare performance estimates directly across rows.

AUC, area under the receiver operating characteristic curve; CE-MS, capillary electrophoresis-mass spectrometry; cfDNA, cell-free DNA; CNV, copy number variation; HR, hazard ratio; LOOCV, leave-one-out cross-validation; MAF, mutant allele frequency; MRD, minimal residual disease; NPV, negative predictive value; OS, overall survival; pCR, pathological complete response; PFS, progression-free survival; RF, random forest; TMB, tumor mutational burden; VAF, variant allele frequency.

### 4.3 Development of a model for recurrence risk stratification and personalized follow-up based on urine multi-omics

This subsection focuses on urinary multi-omics biomarkers for recurrence risk stratification and personalized follow-up. To place urinary biomarkers in clinical context, we briefly refer to tissue-derived molecular subtyping, systemic circulating tumor DNA (ctDNA), clinical risk models, and multimodal integrative frameworks that are not urine-specific. We include these non-urinary approaches only for comparison or context and do not use them to estimate the diagnostic or prognostic performance of urinary biomarkers. Where we discuss such evidence, we explicitly identify it as non-urinary. Developing a multi-omics-based model for recurrence risk stratification and personalized follow-up is a critical step toward achieving precision urological care. In developing risk stratification models, deep integration of clinical and omics data shows tremendous potential. As a non-urinary contextual example, a clinical risk model based on clinicopathological variables—not urinary biomarkers—was developed for patients who underwent radical cystectomy. This model integrated positive urothelial margins, BCG history, carcinoma *in situ*, and preoperative hydronephrosis, and achieved C-statistics of 0.71 and 0.73 for discriminative ability in the training and validation cohorts, respectively, and successfully classified patients into low-, medium-, and high-risk groups, with significant intergroup differences in 5-year survival rates for upper urinary tract urothelial carcinoma (64). This clinical model is cited here to illustrate how risk stratification is currently performed and to highlight the potential complementary role of urinary biomarkers, not as evidence of urinary biomarker performance. At the tissue level, molecular subtypes identified through proteomics and transcriptomics of tumor tissue reflect distinct clinical prognostic features and have been proposed for prognostic stratification (65, 66). These tissue-derived findings are cited here as background to illustrate molecular heterogeneity. They do not constitute evidence for urinary biomarkers and should not be equated with urine-based risk stratification. To further enhance the model’s robustness and clinical applicability, we incorporated multimodal fusion algorithms into model development. For example, the non-urine-specific multimodal framework UroFusion-X integrates 3D imaging, pathology, omics networks, and clinical variables. It is cited here to illustrate methodological advances in multimodal data fusion, not as a urinary biomarker model. Its performance should not be interpreted as evidence for urinary multi-omics diagnostics. Through a cross-modal collaborative attention mechanism, it aligns heterogeneous inputs effectively, maintaining prediction performance above 90% even in real-world scenarios with extensive missing modal data, and showing higher net clinical benefit in receiver operating characteristic (ROC) curve analysis (67). Furthermore, researchers have proposed non-urinary multi-omics regulatory networks integrated with electronic health record data to identify potential biomarkers in large populations. These approaches are cited as contextual examples of data integration. They are not urine-based and do not provide direct evidence for urinary biomarker performance. Through extensive external validation, they identify potential biomarkers applicable to large populations, thereby accelerating the optimization of personalized follow-up strategies and improving patient survival outcomes (68). In conjunction with the principles of risk-stratified guidance for adjuvant therapy and follow-up outlined in the European Association of Urology guidelines, multi-omics-based personalized models will provide more precise decision support for the long-term management of both non-muscle-invasive and muscle-invasive urothelial carcinoma (55, 69). The studies reviewed above demonstrate that urinary multi-omics biomarkers can detect recurrence and stratify risk. Yet, they also reveal a persistent pattern: no single analyte or omics layer consistently outperforms others across all clinical scenarios, and even the most promising panels leave a substantial minority of recurrences undetected. This observation naturally raises the question of whether combining multiple biomarker types—mutations, methylation, proteins, and exosomal cargoes—could overcome each limitation. However, as we will show in the following section, the evidence for additive or synergistic value remains mixed, and the cost-effectiveness of such integrated approaches is largely unexplored. In summary, this subsection primarily evaluates urinary multi-omics biomarkers for recurrence risk stratification. Non-urinary approaches, including tissue-based molecular subtyping, systemic ctDNA, clinical risk models, and multimodal integrative frameworks, are included only as background or comparative context and are not used to support conclusions about urinary biomarker performance.

### 4.4 Challenges posed by false positives and false negatives in the clinical application of single urine omics biomarkers

The main challenges facing single-analyte urinary biomarkers in clinical applications include low sensitivity for early-stage tumors and issues with false negatives and false positives (42). The concentrations of tumor markers in urine are extremely low—sometimes as low as the femtomolar range—which directly contributes to the difficulty of non-invasive diagnosis of bladder cancer (70). For example, among proteomic biomarkers, when CXCL16 and TGFBI proteins in urine supernatant are detected individually, their sensitivities are only 31% and 56%, respectively. Although specificity is high, the extremely low sensitivity leads to many missed diagnoses (71). Regarding nucleic acid testing, conventional circulating tumor DNA (ctDNA) detection methods often miss ultra-short fragments of tumor-derived cell-free DNA in urine. These fragments are typically less than 50 base pairs long, and conventional methods can miss them, leading to significant false-negative results (13). In addition, testing for epigenetic biomarkers also has technical limitations. For example, because changes in 5-methylcytosine and 5-hydroxymethylcytosine are generally negatively correlated, biomarkers based on the traditional bisulfite method that combine these two signals may attenuate cancer-related signals, making them unsuitable as standalone clinical biomarkers (58). For upper urinary tract urothelial carcinoma, the sensitivity of single urine tests such as EpiCheck ranges from 65% to 83%, with specificity from 79% to 83%; the overall level of evidence is low, making these tests difficult to use as standalone diagnostic tools (62). These limitations indicate that biomarkers relying on a single omics dimension struggle to capture the complex molecular heterogeneity of urothelial carcinoma, highlighting an urgent need for an integrated multi-omics strategy to improve diagnostic performance.

### 4.5 Machine learning approaches applied to urinary multi-omics biomarkers in urothelial carcinoma

Faced with the exponential growth of data generated by multi-omics studies, artificial intelligence and machine learning algorithms have become powerful tools for processing, integrating, and interpreting this complex data to extract meaningful insights (72). Researchers have applied machine learning to urinary multi-omics data mainly for feature selection, model construction, and risk classification. In urothelial carcinoma, LASSO regression has been used to select clinical variables and urinary proteomic features for baseline and combined diagnostic models (40, 73). Random forest has been used to detect molecular residual disease from urine cfDNA multi-omics by integrating mutant allele frequency, inferred tumor mutational burden, and copy-number-based tumor fraction (74). Support vector machines have been used to build non-invasive diagnostic models from urinary copy number variation and methylation data, maintaining classification performance at ultra-low sequencing depths (54). A proteomic transfer-learning neural network integrating serum and urine multi-proteomic data has also been reported to improve bladder cancer diagnosis and progression monitoring (75). More complex multimodal frameworks, such as UroFusion-X, integrate imaging, pathology, omics, and clinical variables. However, these are not urine-specific and are cited only as methodological examples (67). Importantly, most machine-learning models in this field remain exploratory, have been developed in single-center or case-control cohorts, and lack external validation. Machine learning may improve data integration, but it does not overcome the need for prospective validation and clinical utility assessment.

## 5 The translational gap: why promising biomarkers have not reached clinical practice

The preceding sections have presented compelling evidence that urinary multi-omics biomarkers can achieve high sensitivity and specificity for urothelial carcinoma detection, often outperforming traditional cytology and rivaling cystoscopy in carefully selected cohorts. However, as discussed below, major international guidelines do not recommend routine use of these tests. This discrepancy —the gap between enthusiastic research reports and conservative clinical recommendations—reflects the field’s current state and forms the core of this review (**Figure 3**).

**Figure 3:**
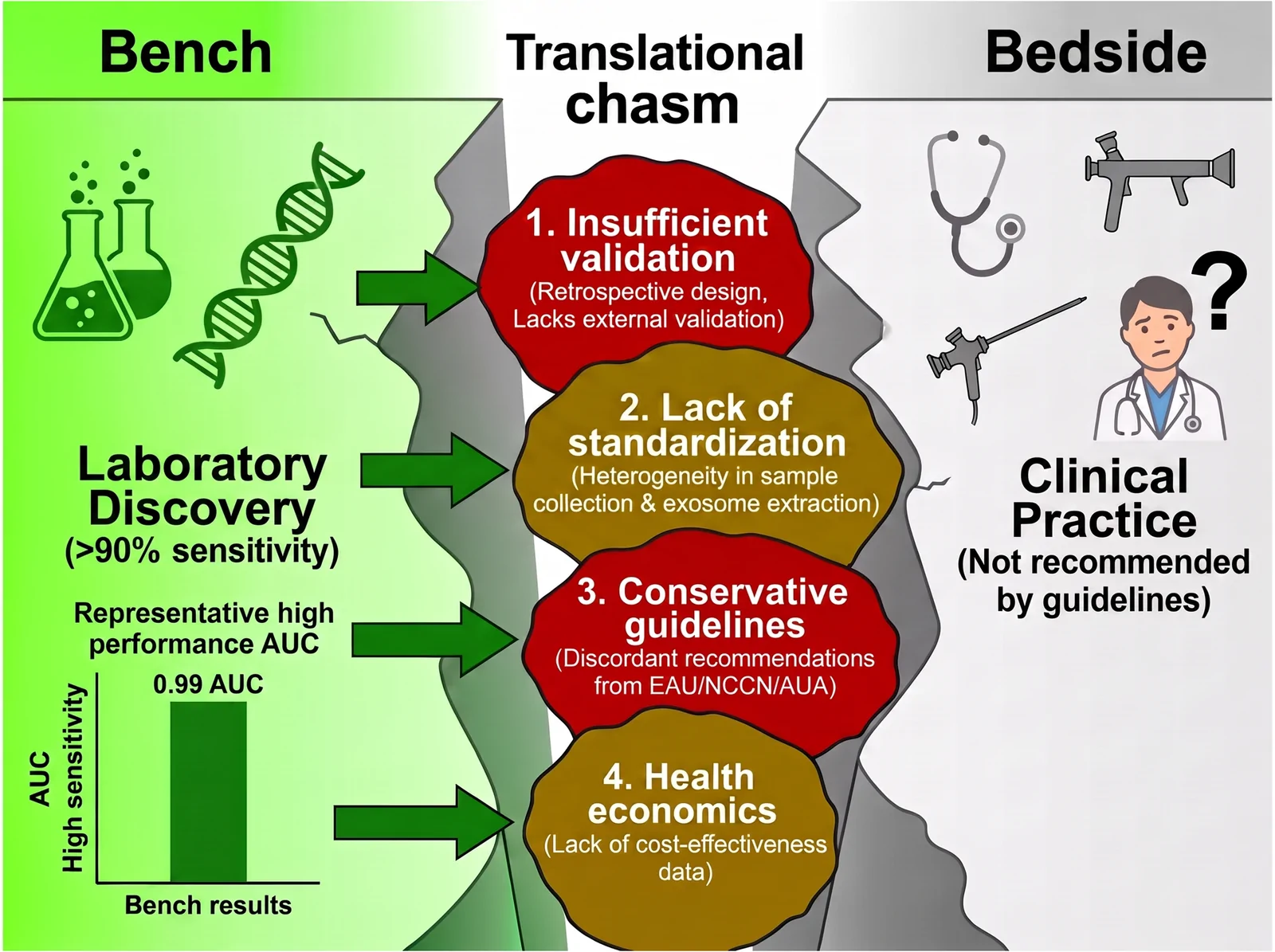
Translational barriers preventing clinical adoption of urinary multi-omics biomarkers. This conceptual illustration depicts the critical gap (“Chasm”) between promising research findings and routine clinical practice. The left side (“Bench”) represents the laboratory setting, where urinary multi-omics panels achieve diagnostic performance exceeding 90% sensitivity and specificity in carefully designed studies. The right side (“Bedside”) represents clinical practice, where cystoscopy remains the gold standard and major guidelines (EAU, AUA, NCCN) do not recommend routine use of urinary biomarkers. Four core translational barriers are shown as obstacles spanning the gap: (1) Limited validation—most studies are retrospective case-control designs with insufficient external validation; (2) Lack of standardization—substantial heterogeneity in sample collection, exosome isolation, and analytical protocols across laboratories; (3) Guideline conservatism—divergent and generally cautious recommendations from major urological societies; (4) Health economic gaps—absence of cost-effectiveness data and reimbursement pathways. This figure provides a visual framework for the critical appraisal presented in Section 5 and reinforces the central argument that diagnostic accuracy does not automatically translate into clinical utility. AUA, American Urological Association; EAU, European Association of Urology; NCCN, National Comprehensive Cancer Network.

### 5.1 Critical appraisal of clinical validation evidence for multi-omics integrated models

Section 3 summarized the encouraging diagnostic performance of urinary multi-omics panels in research settings. Here, we critically appraise the quality of evidence supporting these claims, identifying caveats that temper optimistic interpretations and contribute to the translation gap. The multidimensional integrated model demonstrated significant advantages in validating diagnostic performance for urothelial carcinoma by integrating different types of molecular biomarkers. Regarding the combination of DNA mutations and methylation, the UI-Seek model integrates FGFR3 and TERT gene mutations with ONECUT2 and VIM methylation abnormalities. In a prospective multicenter trial, it showed 91.37% sensitivity and 95.09% specificity, with 75.81% sensitivity for low-grade Ta-stage tumors and over 93% for high-grade tumors (42). Another urine-based genomic test combined mutation status of FGFR3, TERT, and HRAS with methylation status of OTX1, ONECUT2, and TWIST1, demonstrating robust performance across hematuria risk groups, with an area under the ROC curve ranging from 0.929 to 0.971 (76). Regarding pure methylation marker combinations, a diagnostic model incorporating three markers—VIM, TMEM220, and PPM1N—achieved an overall sensitivity of 94.44% and an overall specificity of 93.52% for urothelial carcinoma, with sensitivities of 80% and 88.57% for stage 0a/0is and early stage I lesions, respectively (77). The Bladder CARE index, which analyzes the methylation levels of three tumor markers—TRNA-Cys, SIM2, and NKX1-1—achieved a detection sensitivity of 96% and a specificity of 88%, significantly outperforming traditional urinary cytology (41). In combined protein and methylation analysis, urinary protein biomarkers CXCL16 and TGFBI, along with DNA methylation markers, increased sensitivity for identifying patients with urothelial carcinoma to 72% and specificity to 97% (71). Furthermore, a classifier based on the urinary EV proteome can predict bladder urothelial carcinoma with 92% accuracy by identifying specific protein biomarkers associated with muscularis infiltration, thereby further expanding the scope of multi-omics-based diagnostic approaches (27).

While the diagnostic performance figures summarized above are encouraging, a rigorous appraisal of the clinical validation evidence for multi-omics integrated models reveals several important caveats that temper optimistic interpretation. First, most published studies use retrospective case-control designs, which are known to overestimate diagnostic accuracy compared with prospective screening or surveillance cohorts. Case-control designs enroll patients with established diagnoses and healthy controls, thereby artificially inflating the apparent separation between disease and non-disease groups. The performance metrics reported in such studies—often exceeding 90% sensitivity and specificity—should be interpreted with caution, as they may not generalize to real-world populations with lower pre-test probabilities and more heterogeneous differential diagnoses. Second, external validation, the gold standard for demonstrating generalizability, remains conspicuously lacking for most multi-omics panels. Of the studies cited in this review, only a minority have been validated in independent, geographically distinct cohorts. The UCseek model, for instance, performed well in its derivation cohort, but its performance in multicenter external validation has not yet been fully reported. Even for the few panels with multicenter validation, the participating institutions are often within the same country or region, limiting generalizability to diverse ethnic populations and healthcare settings where disease prevalence and patient demographics differ substantially.

Third, sample sizes in most validation studies are insufficient to provide robust estimates of sensitivity and specificity, particularly for subgroup analysis by tumor stage and grade. Exploratory rather than confirmatory endpoints power many biomarker discovery studies, and confidence intervals around reported performance metrics are often wide, reflecting imprecision that limits clinical confidence. Fourth, the high-performance figures reported in the literature are likely subject to publication bias. Studies with positive findings are more likely to be published, while studies with equivocal or negative results—which may represent a substantial portion of the total research effort—remain unpublished. This is particularly concerning in the biomarker field, where the “winner’s curse” is well-documented. Fifth, direct head-to-head comparisons of different multi-omics panels in the same patient cohort are almost absent. Consequently, it remains unclear whether the UI-Seek panel outperforms the UriMee model, or whether methylation-based approaches are superior to mutation-based panels, in the same clinical setting. Without such comparative data, clinicians and guideline panels struggle to determine which test offers the best value. Finally, the distinction between analytical validation and clinical validation is often blurred. Analytical validation—demonstrating that a test can reliably detect methylation markers or mutations—is necessary but insufficient for clinical adoption. Clinical validation requires prospective studies demonstrating that test results guide clinical decision-making and improve patient-relevant outcomes such as reduced unnecessary procedures, earlier detection of recurrence, or improved survival. Such prospective evidence remains scarce for multi-omics integrated models.

These caveats suggest that while the research foundation is robust, the clinical readiness of multi-omics integrated panels remains lower than the enthusiastic literature might imply. Clinicians and policymakers should recognize that most currently available panels are at the proof-of-concept or early validation stage, not yet at the clinical utility stage. The pathway to clinical adoption requires not only larger, prospective, multicenter validation studies but also pragmatic trials that assess how testing affects clinical decision-making and patient outcomes. Until such evidence accrues, guideline panels will appropriately remain conservative in their recommendations, a stance that, as discussed in Section 3.5, reflects the current gap between research findings and evidence-based clinical practice.

#### 5.1.1 The critical evidence gap: clinical utility versus analytical performance

The preceding critique of study designs (Section 5.1) explains why reported performance figures may be inflated. Still, a more fundamental gap undermines translating multi-omics panels from bench to bedside: the near-complete absence of evidence demonstrating clinical utility, distinct from analytical or clinical performance.

In biomarker evaluation, these three hierarchical levels are often conflated. Analytical validity asks whether a test accurately and reliably measures its intended analyte. Clinical validity asks whether the test accurately identifies patients with the disease or predicts outcomes (AUC). Clinical utility, however, asks the decisive question for regulatory and guideline bodies: Does use of this test lead to improved patient-relevant outcomes, such as reduced morbidity from unnecessary procedures, earlier detection of high-grade recurrence, or improved survival, when integrated into clinical decision-making?

The evidence summarized in this review, while robust for clinical validity, is conspicuously silent on clinical utility. Nearly all published studies are observational, either case-control or prospective cohort designs—that correlate biomarker results with disease status at a single time point. What is critically lacking are prospective, interventional trials that randomize patients to biomarker-guided surveillance versus standard-of-care (e.g., cystoscopy-based) follow-up, and measure patient-centered endpoints. For instance, while the BC-106 panel achieves a negative predictive value >95% and could theoretically reduce unnecessary cystoscopies (57), no prospective study has shown that implementing this panel reduces the total number of cystoscopies, lowers complication rates, or improves quality of life in a real-world surveillance population. Similarly, although the UCseek model detects recurrence with higher accuracy than cystoscopy in a monitoring cohort (54), it remains unclear whether earlier molecular detection enabled by this test translates into a survival benefit or reduces the need for radical surgery.

This evidence gap is not merely academic—it has direct consequences for guideline development. The EAU, AUA, and NCCN guidelines explicitly grade recommendations based not only on study design (e.g., prospective vs. retrospective) but also on the availability of data demonstrating net health benefit. The AUA’s cautious endorsement of urinary biomarkers for adjudicating equivocal cytology, but not for replacing surveillance cystoscopy, reflects this distinction: the former is a low-risk, adjunctive use with a lower threshold for clinical utility, whereas the latter would require definitive proof that biomarker-guided surveillance does not compromise oncological safety. Currently, no urinary multi-omics panel has been evaluated in a randomized controlled trial with an oncological safety endpoint, such as time to progression or bladder cancer-specific survival. Until such trials are conducted and published, guideline panels will appropriately regard these promising biomarkers as tools with unproven clinical utility, regardless of their impressive AUCs or sensitivities.

This distinction—between what a test can detect and what a test achieves in clinical practice—represents the single most important barrier to adoption and is the primary reason why the translational chasm (**Figure 3**) persists. Addressing this gap does not require abandoning the field. Still, it does demand a shift in research priorities toward pragmatic, interventional study designs that directly answer the question: Do urinary multi-omics tests make a meaningful difference to patients?

### 5.2 Diagnostic advantages and potential health economic implications of integrated models

Integrated multi-omics models have shown improved diagnostic performance in some studies and may have potential health economic implications. For example, combining a baseline clinical model with a proteomics model increased the AUC from 0.75 to 0.83, and proteomic biomarkers emerged as independent risk factors for longitudinal cancer progression (73). In patients with hematuria, an integrated diagnostic model provided posterior probability estimates: a negative urine test corresponded to a 0.3–2% probability of urothelial carcinoma, whereas a positive test corresponded to 31–42%, which may help supplement current hematuria risk stratification (76). In terms of potential health economic implications, combined models may offer practical and potentially lower-cost testing options, but formal cost-effectiveness data remain lacking. For example, both the utLIFE-UC model and the UCseek model have been described as non-invasive, rapid, and potentially lower-cost testing options. UCseek maintained diagnostic performance at ultra-low sequencing depths in its derivation cohort, which may reduce per-test costs (52, 54). However, formal cost-effectiveness analyses are lacking. In postoperative follow-up management, multi-omics integrated models can significantly reduce unnecessary invasive examinations. Studies have shown that the BC-106 urinary proteomic peptide panel, a multi-marker proteomic assay, when combined with cytology, has a negative predictive value exceeding 95% and has been proposed as a strategy to reduce the frequency of cystoscopy during follow-up (57). Whether this approach is safe and effective in routine practice requires prospective validation. Similarly, UCseek achieved an accuracy rate of 90.91% in a recurrence monitoring cohort, higher than cystoscopy’s 59.09% accuracy in that cohort (54). Whether this translates into improved clinical outcomes, resource use, or cost-effectiveness remains unproven. In the study cohort, decision curve analysis suggested that a multimodal integrated framework yielded higher net clinical benefit than a single-modality baseline (67). This finding requires external validation and formal health economic evaluation before making broader claims. The superior diagnostic performance of integrated multi-omics models, while encouraging, raises a critical question: if these tests are so accurate, why have they not been widely adopted? The answer lies in a constellation of translation barriers that we examine next.

### 5.3 The guideline conundrum: conservatism amidst promising data

Perhaps the most telling indicator of the translational gap is the cautious stance adopted by major international clinical guidelines. Despite the encouraging diagnostic performance reported in numerous studies, a systematic comparison of the most recent guidelines from the European Association of Urology (EAU), American Urological Association (AUA), and National Comprehensive Cancer Network (NCCN) reveals that none of the three recommends the routine use of urinary biomarkers for diagnosis or surveillance of urothelial carcinoma (78).

The EAU guidelines adopt the most conservative stance, advising against routine use of urinary molecular markers and explicitly stating that they “cannot replace cystoscopy in routine practice,” citing limited evidence and concerns regarding cost-effectiveness. The EAU guidelines on NMIBC and UTUC have expanded the section on urinary molecular markers with new data on multiplex urine tests in surveillance. Still, the overall recommendation level remains cautious (55, 69). For upper tract urothelial carcinoma, the EAU Guidelines Panel’s systematic review assigned low certainty to RNA-based panels and DNA methylation panels, underscoring the gap between research findings and guideline endorsement (62).

The AUA guidelines allow selective adjunctive use of validated urinary biomarkers in narrowly defined scenarios: assessing response to intravesical BCG therapy, adjudicating equivocal cytology results (79, 80). The 2025 Microhematuria Guideline amendment also supports risk-stratifying patients with low- to intermediate-risk microscopic hematuria using validated urine-based genomic tests (81). However, the AUA explicitly states that urinary biomarkers should not be used in place of cystoscopic evaluation during NMIBC surveillance.

The NCCN guidelines have shown the most notable evolution in this space. The most recent NCCN guidelines incorporated urinary liquid biopsy into routine screening for high-risk patients, whereas earlier versions had limited this recommendation to recurrence monitoring (82). Additionally, NCCN recommends ctDNA-MRD testing for risk stratification and adjuvant immunotherapy decision-making in muscle-invasive bladder cancer. Nevertheless, for low-risk NMIBC patients, NCCN still considers that urinary cytology and other urine biomarker tests have “no routine application value” (82).

The divergence among guidelines, particularly the EAU’s conservative stance versus the NCCN’s gradual embrace of liquid biopsy in specific high-risk contexts, reflects differences in regional regulatory environments, the pace of evidence integration, and varying thresholds for adopting novel technologies (78). What unites them, however, is the consensus that current evidence is insufficient to support replacing cystoscopy with any urine-based test. This guideline-level conservatism serves as a crucial counterbalance to the enthusiastic reports in the research literature, reminding clinicians and researchers that diagnostic accuracy in well-controlled studies does not automatically translate into clinical utility in unselected, real-world populations.

### 5.4 Development of standard operating procedures for sample collection, processing, and storage

The complexity of urine samples requires strict standard operating procedures to ensure reliable multi-omics data. Liquid biopsy may provide serial information about changes in the tumor microenvironment by analyzing components such as circulating extracellular vesicles; its separation techniques—including microfluidics, magnetic beads, and aptamers—can be combined with high-throughput multi-omics analysis to identify biomarkers that predict treatment response and recurrence (83).

However, the mechanisms underlying the secretion and delivery of markers such as exosomal circular RNA remain unclear, and detection methods require further refinement, directly limiting clinical translation (50). In proteomics, commercial high-throughput platforms enable integrated multi-omics measurements in large-scale epidemiological studies; however, data quality issues remain underestimated, and platform-specific problems may lead to inconsistent results. Therefore, absolute or reliably calibrated relative quantitative methods are needed to guide patient management, and orthogonal validation is required (84).

Furthermore, microsampling technology combined with wearable sensors has enabled the frequent capture and analysis of thousands of metabolites, lipids, cytokines, and proteins in as little as 10 microliters of sample. This high-frequency microsampling approach provides new technical insights for the standardized collection and dynamic monitoring of urine samples (85). Establishing standard operating procedures (SOPs) covering the entire process—from sample collection, processing, and storage to nucleic acid or protein extraction is essential to eliminate batch effects and ensure data comparability across multicenter studies (86).

## 6 Summary and outlook: a look ahead at the accumulation and translation of findings on urinary multi-omics biomarkers

This article systematically reviews progress on multi-marker urinary biomarkers for the non-invasive diagnosis, postoperative monitoring, and clinical translation of urothelial carcinoma, clarifying their value in replacing traditional invasive tests and identifying future development directions.

### 6.1 Summary of key research findings

This review systematically examined the landscape of urinary multi-omics biomarkers for urothelial carcinoma, focusing on the translational gap between research findings and routine clinical adoption. Our analysis yields four main conclusions. First, at the technical level, urinary cfDNA/methylation, miRNAs, exosomes, and proteomic/metabolomic signatures have each demonstrated robust analytical performance, with integrated multi-omics models consistently outperforming single-analyte approaches. Models such as UI-Seek (an integrated genomic–epigenomic assay) (42), UriMee (a multi-marker methylation model) (40), and UCseek (integrated CNV–methylation profiling) (54) have achieved sensitivity and specificity exceeding 90% in their respective study cohorts. Notably, the H4C6/TWIST1 two-gene methylation assay achieved 93.02% sensitivity and 92.13% specificity in a prospective, multicenter study of 743 urine samples (39). However, these performance figures derive from carefully designed studies—including prospective diagnostic accuracy studies and model development–validation designs—with selected patient and control groups. They may not generalize directly to unselected real-world screening populations.

Second, in postoperative monitoring and recurrence prediction, urinary biomarkers offer promise because they are non-invasive and repeatable. The BC-106 panel combined with cytology achieved a negative predictive value exceeding 95%, suggesting potential to reduce unnecessary cystoscopies during follow-up (57). However, the evidence base remains strongest for early detection and weakest for guiding treatment decisions, highlighting priority areas for future research.

Third, as discussed in Section 5, major clinical guidelines (EAU, AUA, NCCN) remain conservative, citing persistent evidence gaps, including limited external validation, the absence of head-to-head comparisons, and a lack of cost-effectiveness data.

Fourth, closing the translational gap will require coordinated efforts across multiple domains: technical standardization and cost reduction; prospective, pragmatic trial designs with embedded health-economic analysis; regulatory and reimbursement pathways that incentivize innovation while protecting patients; and clinician education to facilitate appropriate interpretation and integration of multi-omics reports.

### 6.2 Future directions

Looking forward, we envision a phased pathway for the clinical integration of urinary multi-omics diagnostics. In the near term, priority should be given to establishing standardized pre-analytical protocols and reference materials and prospectively validating leading panels in unselected populations. The clinical adoption of urinary biomarkers is most realistic in specific, well-defined scenarios: adjudicating equivocal cytology results, stratifying surveillance intensity in NMIBC patients, and assessing BCG response—areas where the AUA guidelines already permit selective adjunctive use (81, 87).

In the medium term, we anticipate the emergence of commercially available, FDA-cleared or CE-marked multi-omics platforms with clear indications for use. The NCCN’s gradual embrace of urinary liquid biopsy in high-risk patients may signal a broader shift toward guideline-endorsed adoption, contingent on generating higher-level evidence from biomarker-driven trials (82).

In the long term, urinary multi-omics diagnostics may become an increasingly valuable component of the clinical pathway for urothelial carcinoma, integrated into routine surveillance algorithms and potentially guiding molecularly targeted therapy through transcriptomic and proteomic subtyping. However, even with continued progress, these tests are most realistically positioned as complementary decision-support tools that may guide more selective use of cystoscopy and inform risk-stratified surveillance, rather than replacing established diagnostic algorithms.

## 7 Conclusion

In conclusion, urinary multi-omics biomarkers offer substantial promise as non-invasive, repeatable tools that capture tumor heterogeneity, but, based on current evidence, their limitations—variable sensitivity in low-grade disease, lack of standardized protocols, and false-negative risks—preclude their use as replacements for cystoscopy, which remains the cornerstone of diagnosis and surveillance, particularly in NMIBC. Their most realistic role today is complementary decision-support aids in selected scenarios, while the aspirational goal of non-invasive, precise, patient-centered care awaits sustained multidisciplinary effort.
